# Residual varus alignment after posterior-stabilized total knee arthroplasty limits medial soft tissue remodeling

**DOI:** 10.1186/s12891-023-07048-8

**Published:** 2023-11-28

**Authors:** Yanfeng Jia, Leilei Zhai, Shiqi Qin, Juncai Xu, Wei Gao, Boxuan Zhang, Xiaofeng Wang, Kezhen Zhou, Zhiwen Sun, Yingzhen Niu, Hongwei Bao, Ran Sun

**Affiliations:** 1 Department of Orthopaedic Surgery, Jingjiang People’s Hospital, Taizhou, Jiangsu, 214500 China; 2 Department of Orthopaedic Surgery, Third Hospital of Hebei Medical University, Shijiazhuang, 050051 Hebei China; 3 Center for Joint Surgery, Southwest Hospital Army Medical University, Chongqing, 400038 China

**Keywords:** Total knee arthroplasty (TKA), Varus–valgus laxity, Lower limb alignment, Varus knee

## Abstract

**Background:**

Proper lower limb alignment and soft tissue balance are significant indicators to measure the success of total knee arthroplasty (TKA). Previous studies have confirmed that soft tissue relaxation around the knee after TKA will change over time; however, the relationship between lower limb alignment and soft tissue balance after TKA remains unclear. We studied (1) whether the change of soft tissue balance around the knee with time after posterior-stabilized (PS) TKA would affect the alignment of the lower limbs; (2) Whether the accuracy of lower limb alignment during PS TKA affects postoperative soft tissue remodeling.

**Methods:**

In this study, 100 patients were recruited after PS TKA. Among them, 50 patients with a hip knee ankle (HKA) angle of ≤  ± 3° were set as the neutral group, and 50 patients with an HKA angle of >  ± 3° were set as the deviation group. The imaging results measured the HKA angle before the operation as well as the HKA, varus, and valgus angles at 1, 3, 6, 12, and 24 months after TKA. Clinical assessment included range of motion (ROM), Western Ontario and McMaster Universities Osteoarthritis Index (WOMAC), and Knee Society Score (KSS).

**Results:**

Eight people were excluded from the study. After the exclusion, the study enrolled 47 patients in the neutral group and 45 patients in the deviant group and were followed for up to 2 years. There was no statistical significance in mean varus angles as well as HKA angle changes during the follow-up phase of each groups (*P* > 0.05). The mean valgus angles of the patients in the neutral group group were 2.47°, 3.45°, 3.63°, 3.60° and 3.63°, and in the deviation group were 2.45° (*P* = 0.841), 2.88° (*P* < 0.001), 3.07° (*P* < 0.001), 3.06° (*P* < 0.001), and 3.10° (*P* < 0.001). ROM, WOMAC and KSS of the two groups were significantly improved after operation, with no difference between the two groups.

**Conclusion:**

This study shows that whether the alignment is accurate or not in the early stage after TKA, the relaxation of the medial and lateral soft tissues of the knee joint change; however, this change will not significantly affect the alignment of the lower limbs. Postoperative residual varus deformity limits medial soft tissue remodeling.

**Level of evidence:**

III.

## Introduction

Total knee arthroplasty (TKA) is considered to be the most effective way to improve the quality of life of patients with severe knee osteoarthritis. Surgeons attribute great importance to TKA alignment correction and achieve neutral alignment through intraoperative osteotomy and soft tissue balance. Although the surgical technique has been very mature, errors inevitably occur during the surgical operation to correct alignment. Theoretically, the primary factor affecting the lower limb mechanical force line after surgery is the change of soft tissue. So far, few reports have studied the effect of soft tissue balance on the lower limb force line. The relaxation of the medial and lateral joint space represents the state of soft tissue balance. Previous studies have shown that slight joint space relaxation after TKA is acceptable [1–3]. Since abnormal movement after TKA leads to increased early and long-term wear of polyethylene and poor prognosis, proper alignment of the limbs and the components achieved via accurate soft tissue balance is considered to be one of the most important considerations for a successful TKA [3]. Excessive stretching of the soft tissue of the knee joint may lead to joint pain, stiffness, and reduced range of motion, while excessive relaxation of the soft tissue is related to joint instability, accelerated polyethylene wear and surgical failure.

Various surgical techniques and balance devices have been reported in previous studies, which can achieve an appropriate gap balance from extension to deep flexion [4]. Soft tissue balance can also be accomplished by balancing the joint space and making each space rectangular; however, it is challenging for the operator to make the joint space reach the perfect rectangular shape during the operation. A large number of previous studies have reported that healthy knees have physiological lateral relaxation; therefore, slight lateral relaxation after TKA is normal [5]. The long-term prognosis after TKA is good, and the lateral relaxation is approximately 5°, which is similar to that of healthy knees [6]. Previous studies have also shown that the soft tissue balance after TKA changes spontaneously over time [2, 7]. However, the relationship between the soft tissue changes after TKA and the lower limb force line remains unclear. Furthermore, few studies have specifically studied the optimal soft tissue relaxation of a PS TKA [8].

The purpose of this study is to evaluate the degree of change of the internal and external soft tissue relaxation of patients after the initial PSTKA operation over time and whether the change has an impact on the lower limb force line, and to study whether the residual lower limb varus deformity after the operation will have an impact on soft tissue remodeling. The hip, knee, and ankle (HKA) angle in the weight bearing position represents the lower limb force line. Previous studies have proven that after TKA, varus within 3° represents a neutral state [9, 10]. The included angle of the mechanical femoral tibial axis (MFTA) is the included angle between the parallel line of the tibial component platform. The line connecting the lowest point of the medial and lateral condyles of the femoral component, represents the soft tissue relaxation after TKA. This study assumes that the soft tissue around the knee joint will be remodeled early after TKA and affects the lower limb force line. The degree of ligament change is related to the lower limb force line before TKA and the soft tissue operation during TKA. In this study, MFAT and HKA were evaluated in stress and standing radiographs of patients 1, 3, 6, 12, and 24 months after TKA. The ligament tension after TKA was evaluated using the knee ligament gauge.

## Materials and methods

### Patient selection

From May 2019 to November 2020, 100 patients with PS TKA (DePuy PS150) performed by a senior doctor in our hospital were selected. The inclusion criteria of this study are as follows: 1. Osteoarthritis patients with varus deformity 2. Patients with initial PSTKA 3. The age of the patients is between 50 to 80 years old. During the study period, the exclusion criteria were applied and included the following: 1. Patients with severe knee joint deformity or severe bone defect that needed a bone graft. 2. Other types of arthritis, such as rheumatoid, psoriatic, and inflammatory arthritis. 3. The patient has been diagnosed with the following disease states: neuropathic pain or osteonecrosis of the femoral head or hip arthritis. 4. The patient has undergone other joint surgery except knee joint surgery before or during follow-up.

Based on the inclusion and exclusion criteria, a total of 92 knees were included in this study. We divided the groups according to the mechanical alignment during the first X-ray examination of the lower limbs in a standing position after surgery.

Forty-seven patients with PS TKA whose HKA angles were ≤ ± 3 ° at 1 month after surgery were selected in neutral group, and 45 patients with PS TKA whose HKA angles were >± 3 ° at 1 month after surgery were selected in deviation group. Baseline characteristics and demographic details for all study groups are shown in Table 1. Informed consent was obtained from all study participants. Institutional review board approval was obtained before study commencement.

**Table 1:** The demographics and preoperative clinical data of the patients

	Neutral group	Deviation group	*P* value	All people
Knees (n)	47	45	-	92
Sex (female /male)	36/11	36/9	-	72/20
Age	65.60 ± 5.66	65.73 ± 4.93	0.90	65.66 ± 5.29
Follow-up period (months)	24.52 ± 0.40	24.39 ± 0.36	0.11	24.46 ± 0.38
BMI (kg/m2)	27.28 ± 3.49	27.66 ± 2.85	0.57	27.47 ± 3.18

Data are shown as mean ± SD

*BMI* body mass index

### Surgical technique

All surgeries were performed by a single experienced surgeon. All joints were exposed using the medial parapatellar approach, and the measured resection technique was used for bone cutting. The posterior femoral osteotomy was performed under the condition of knee joint flexion, the surgical TEA was drawn, and the natural PCA was measured with a goniometer having a measurement accuracy of 1° and installed on the resection guide. The same experimental tibial spacer used for the extension gap was placed in the flexion gap, and any remaining lateral space was filled with a 1 mm spacer until the lateral soft tissue was at its maximum tension, making it possible to estimate the asymmetry of the initial flexion gap. The patella was not replaced in all cases of knee arthroplasty, only the patellar osteophyte was removed, and the balance of soft tissue was adjusted.

All patients received identical pain control and rehabilitation after operation, among which the multimodal method was designed to avoid injecting anesthetic and advance the patient's postoperative activity time.

### Radiographic evaluation and measurement of soft tissue laxity

**Fig. 1:**
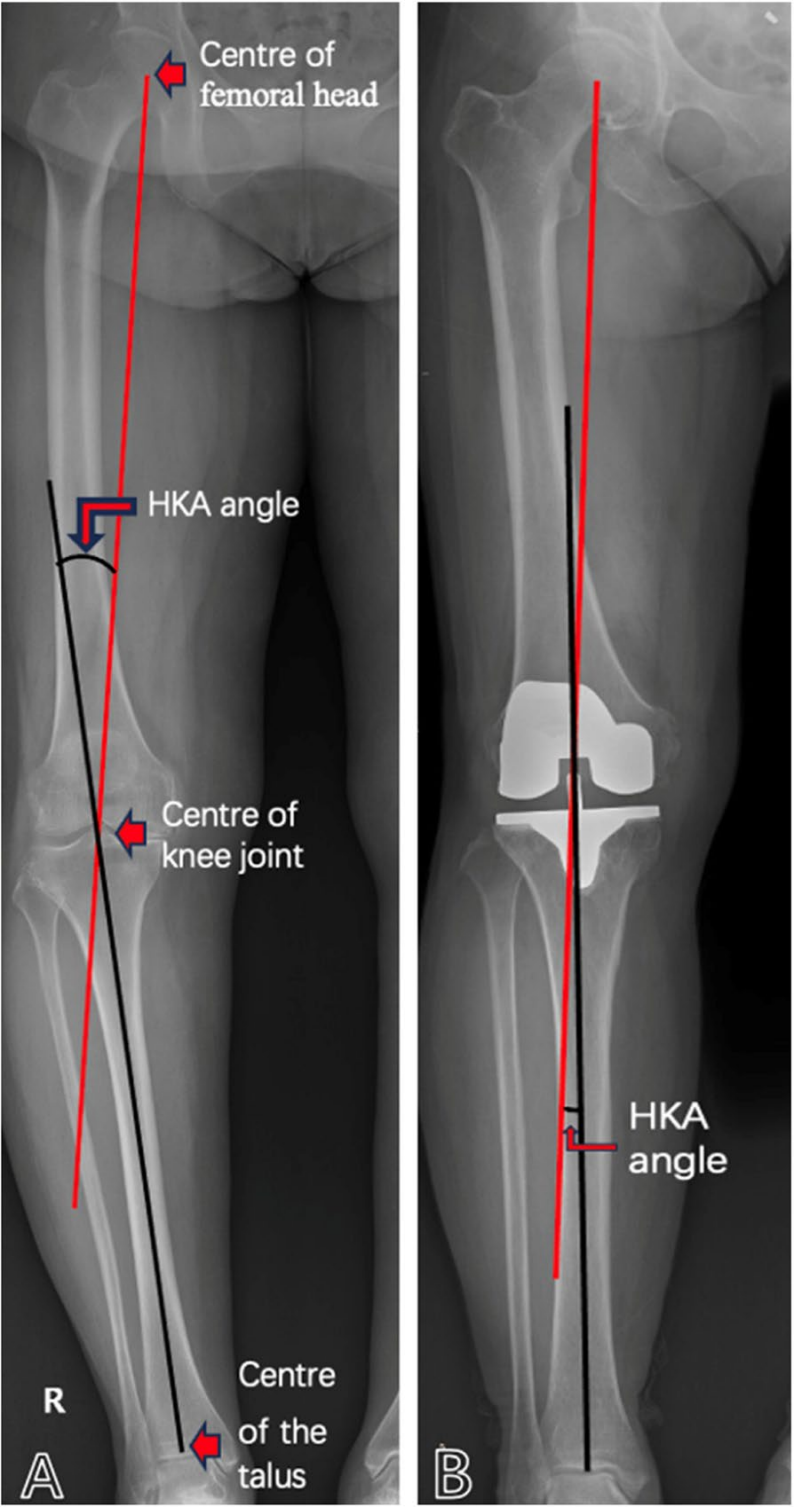
The hip knee ankle (HKA) angle represents the alignment degree of the lower limb, and is the included angle formed by the line from the midpoint of the femoral head to the midpoint of the knee joint and the line from the midpoint of the talus to the midpoint of the knee joint. **A**: Postoperative HKA angle. **B**: Preoperative HKA angle

The template with concentric circles can be used to determine the center of the femoral head. The midpoint of the knee before surgery is defined as the intersection of the midline between the tibial spine and the connecting line between the medial and lateral femoral condyles. The midpoint of the knee after surgery is defined as the intersection of the connecting line between the medial and lateral femoral condyles of the femoral prosthesis and the perpendicular line at the midpoint of the tibial platform. The middle of the talus is defined as the midpoint of the ankle joint. The line from the center of the femoral head to the center of the knee is defined as the mechanical femoral shaft. The line from the center of the knee to the center of the ankle joint is defined as the mechanical tibial axis (Fig. 1).

We performed a radiologic examination on the subjects 1 day before operation and during the follow-up after operation. All patients underwent X-ray imaging of the lower limb alignment including the hip, knee, and ankle joints (the patient was in a standing position and did not wear shoes. The tibial tubercle was in a forward position).

**Fig. 2:**
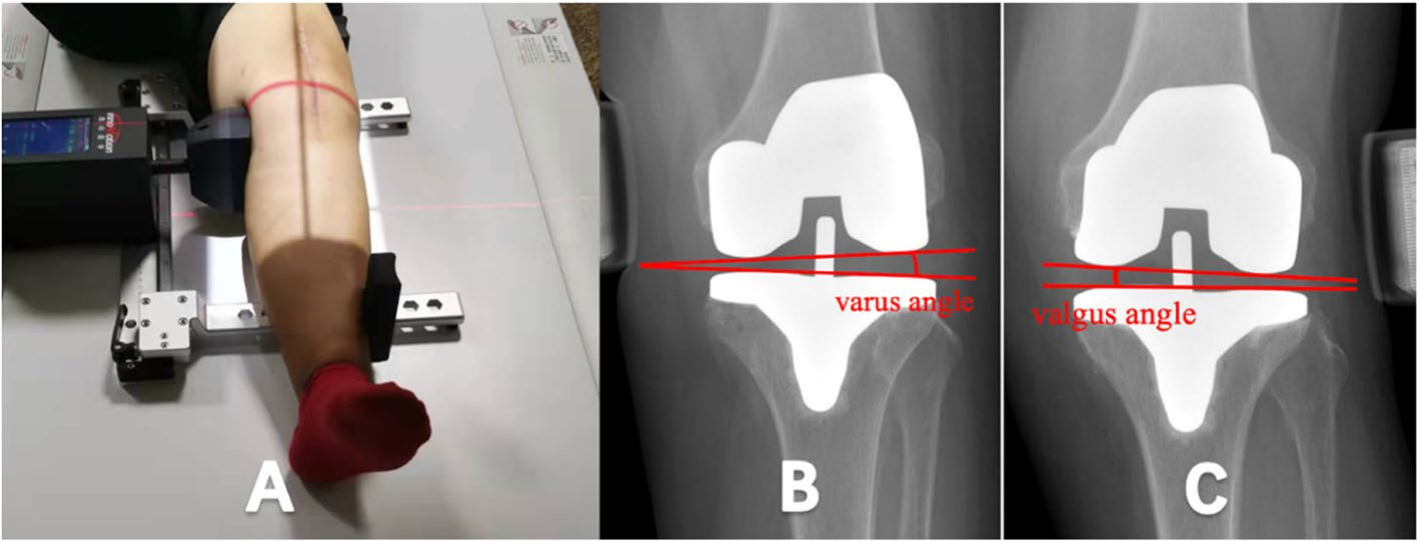
**A**: X-ray of stress position taken when 100N force is applied with knee joint ligament measuring device. **B**: The measurement of the varus angle after TKA is the included angle formed by the line from the lateral femoral condyle to the medial femoral condyle and the parallel line of the tibial plateau. **C**: The measurement of the valgus angle after TKA is the included angle formed by the line from the medial condyle of the femur to the lateral condyle and the parallel line of the tibial plateau

The pre and postoperative HKA angles were measured on full-length weight-bearing coronal radiographs. We use the HKA of 0° on the coronal radiograph to represent the standard neutral position. The patient was in the supine position for stress photography, and the knee was in a straight position during the inspection to minimize the impact of flexion contracture on the results. The standardized technology was used to apply 100N varus and valgus loads to the knee using the knee ligament measuring device (Fig. 2a). The reason for choosing this traction tension is that it can accurately open the internal and external joint space when the knee is fully extended, with many subjects being able to bear this force [11]. The varus and valgus angles were measured on the stress X-ray photograph. When the stress is applied to the inside of the knee joint, the included angle formed by the connecting line from the lateral femoral condyle to the medial condyle and the parallel line of the tibial platform is the varus angle (Fig. 2b). When the stress is applied to the outside of the knee joint, the included angle formed by the connecting line from the medial femoral condyle to the lateral condyle and the parallel line of the tibial platform is the valgus angle (Fig. 2c).

### Patient-Reported outcome scores

Two researchers used a 2-arm goniometer to measure the knee joint range of motion (ROM) of patients pre- and post-operative. ROM is the value of knee joint flexion angle minus the straightening angle. Patient questionnaires under the supervision of the research scientist were then used to record and measure the Western Ontario and McMaster Universities Arthritis Index (WOMAC) and Knee Society Score (KSS) at all periods.

### Patient follow-up

During the recruitment period, 100 patients participated in the study. The average age was 65.66 years (55 to 79 years), and the average body mass index (BMI) was 27.47 kg/m2 (20 to 39). At the last follow-up, 8 people were lost to follow-up (3 in the neutral group and 5 in the deviation group), 2 of the patients withdrew because of knee pain caused by the application of 100n load during stress photography, 1 case lost to follow-up, and 5 cases had unqualified imaging data. Finally, 92 knees participated in this study.

### Statistical analysis

The data differences between the two groups before surgery were compared using an independent sample t-test. The data differences between the groups pre- and post-operation were analyzed using a multivariate analysis of variance and multiple comparisons, and the ROM differences were analyzed via a rank sum test, with *P* < 0.05 defined as statistically significant. Inter-observer reliability was determined by two observers performing the same measurements and intra-observer reliability by having the same observer measure the parameters twice, with an interval of 1 month. Intraclass correlation coefficients (ICCs) were calculated using a SPSS (version 26.0 for Windows; IBM) two-way random model. Interobserver correlation coefficient values were high for all study outcomes recorded.

## Results

A total of 92 patients with PSTKA were included, including 47 in the neutral group and 45 in the deviation group. The average follow-up time of the neutral group was 24.52 months, and the deviation group was 24.39 months (Table 1).

There was no significant difference in HKA angle (mean 9.57° in the neutral group and 9.74° in the deviation group) as well as the ROM between the two groups before operation. There was no statistically significant change of patients HKA angle in neutral and deviation groups during the follow-up post-operation (Table 2). The ROM of the two groups of patients pre- and post-operation at the last follow-up are shown in Table 3.

**Table 2:** Two groups of HKA angle data

Category	1 month	3 month	6 month	12 month	24 month	Multiple comparison
Neutral group HKA	1.64 ± 0.82	1.66 ± 0.81	1.69 ± 0.83	1.67 ± 0.83	1.64 ± 0.80	*p* > 0.05
Deviation group HKA	4.35 ± 0.62	4.38 ± 0.62	4.40 ± 0.61	4.42 ± 0.63	4.42 ± 0.62	*p* > 0.05

There was no statistical difference between the two groups in terms of the HKA angle at each time period postoperative (*p* > 0.05)

*HKA* Hip knee ankle

**Table 3:** Pre- and post-operative KSS, WOMAC, ROM and category

Functional measure	category	Neutral group	Deviation group	*P* value
m				
KSS		36.61 ± 9.22	36.89 ± 9.37	0.889
WOMAC	Total	34.71 ± 5.00	33.07 ± 6.19	0.165
	Pain	35.15 ± 3.89	34.02 ± 5.63	-
	Function	30.89 ± 5.10	30.24 ± 5.72	-
	Stiffness	38.11 ± 8.73	34.96 ± 8.52	-
ROM	angle (°)	86(83^~^94)	86(81^~^93)	0.627
Post-operation				
KSS		86.19 ± 5.72^a^	87.60 ± 4.51^a^	0.194
WOMAC	Total	73.08 ± 5.72^a^	72.72 ± 4.49^a^	0.738
	Pain	78.11 ± 5.48^a^	77.20 ± 5.35^a^	-
	Function	68.91 ± 5.63^a^	69.82 ± 5.16^a^	-
	Stiffness	72.21 ± 7.40^a^	71.13 ± 4.45^a^	-
ROM	angle (°)	118(107^~^124)^a^	118(106^~^125)^a^	0.873

ROM are expressed as median(upper quartile, lower quartile), other data are shown as mean ± SD

Neutral group: KSS *P* < 0.01; WOMAC *P* < 0.01; ROM flexion *P* < 0.01; ROM extension *P* < 0.01

Deviation group: KSS *P* < 0.01; WOMAC *P* < 0.01; ROM flexion *P* < 0.01; ROM extension *P* < 0.01

*KSS* Knee Society Score, *WOMAC* Western Ontario and McMaster Universities Osteoarthritis Index, *ROM* range of motion

^a^were significantly improved than that parameter pre-operation

**Fig. 3:**
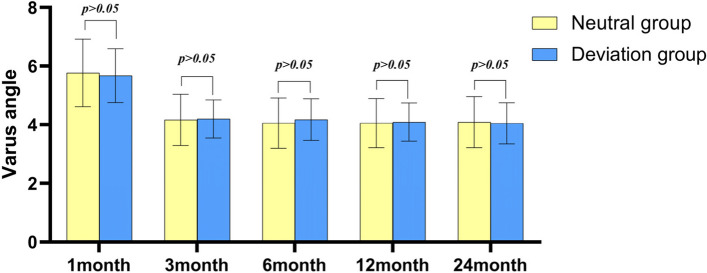
Barrel chart of varus angle at different time after TKA grouped by lower limb alignment (yellow = neutral group, blue = deviation group). The error is expressed by standard deviation. There was no significant difference between groups at all time periods

**Fig. 4:**
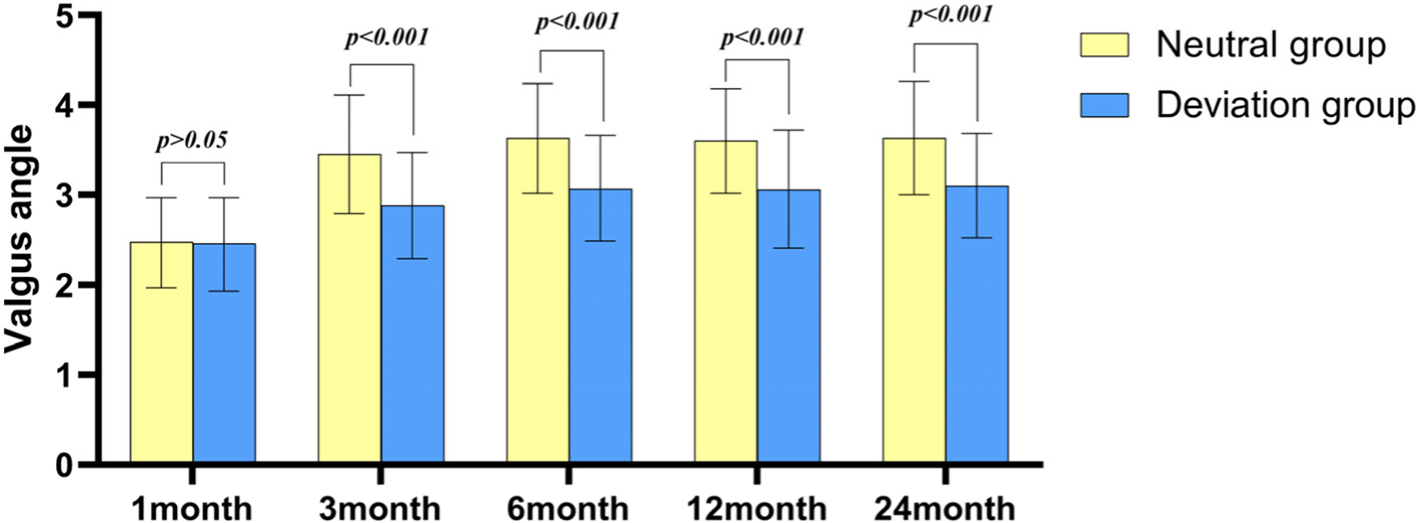
Barrel chart of valgus angle at different time after TKA grouped by lower limb alignment (yellow = neutral group, blue = deviation group). The error is expressed by standard deviation. There was no significant difference between the two groups at 1 month after operation, but there was significant difference between the two groups at 3, 6, 12 and 24 months after operation (*p* < 0.001)

**Fig. 5:**
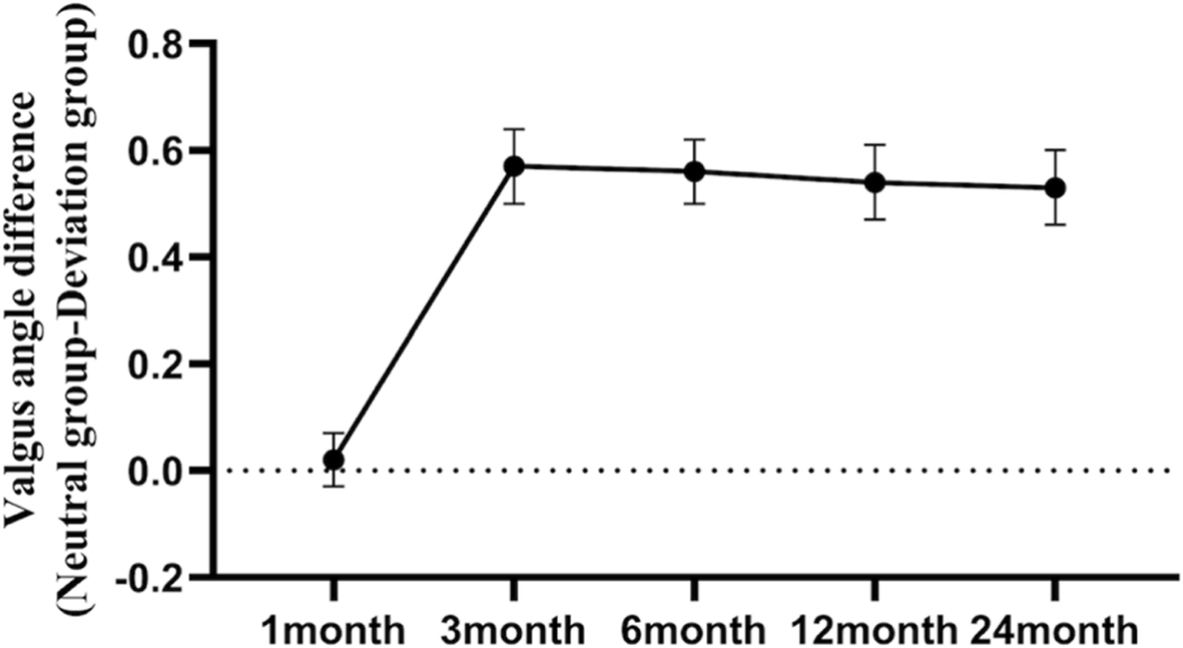
The line chart of the difference of valgus angle between the neutral group and the deviation group at each follow-up after total knee arthroplasty

After operation, the knee varus angle represents the lateral soft tissue relaxation, and the valgus angle represents the medial soft tissue relaxation. During the follow-up, the average varus angles of patients in the neutral group and deviation group was not statistically difference (Fig. 3). The mean valgus angles of the patients was not significantly different between the two groups at 1 month after surgery (Fig. 4, Table 4). At 3, 6, 12 and 24 months post-operation, the average valgus angle difference between the neutral and the deviation groups was 0.57°, 0.56°, 0.54° and 0.53°, respectively, and was statistically significant (Fig. 5).

**Table 4:** Data of varus angle and valgus angle in two groups postoperative

Result Category	Neutral group	Deviation group	*P* value
Varus-angle			
1 month	5.77 ± 1.15	5.68 ± 0.92	0.68
3 month	4.17 ± 0.87	4.20 ± 0.65	0.875
6 month	4.06 ± 0.86	4.18 ± 0.70	0.441
12 month	4.06 ± 0.84	4.09 ± 0.65	0.853
24 month	4.09 ± 0.87	4.05 ± 0.70	0.796
Valgus-angle			
1 month	2.47 ± 0.50	2.45 ± 0.52	0.841
3 month	3.45 ± 0.66	2.88 ± 0.59	0
6 month	3.62 ± 0.61	3.07 ± 0.59	0
12 month	3.60 ± 0.58	3.06 ± 0.66	0
24 month	3.63 ± 0.63	3.10 ± 0.58	0

Data are shown as mean ± SD

**Fig. 6:**
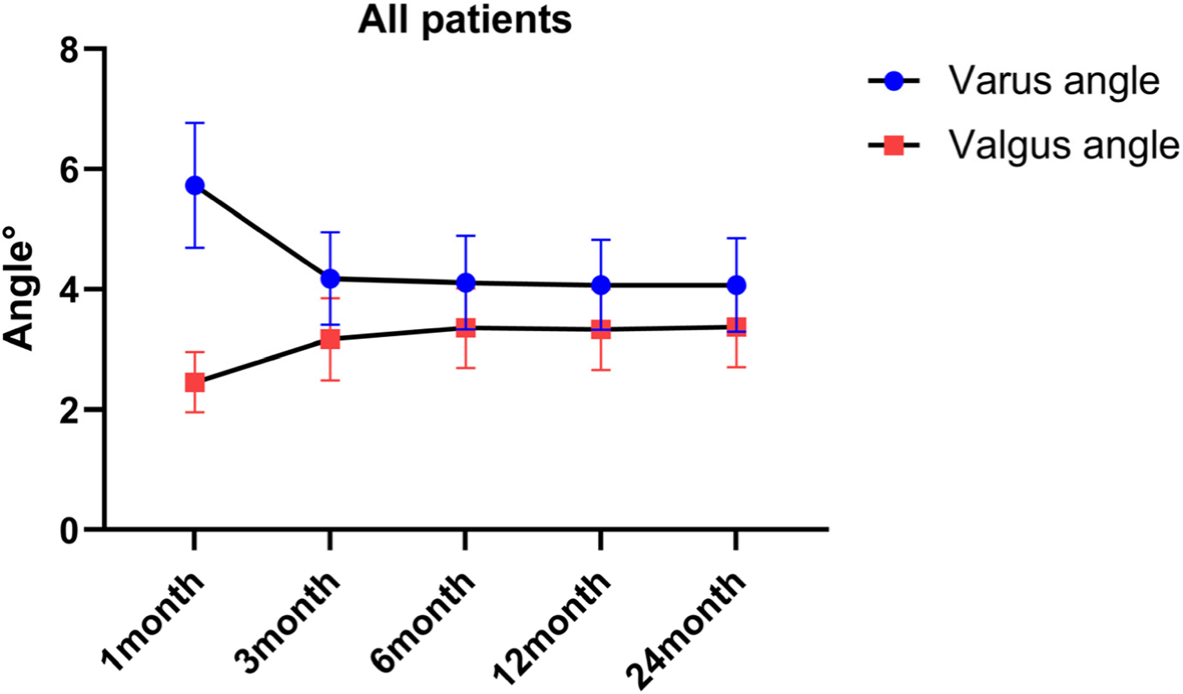
The line chart of varus angle and valgus angle at each follow-up of all patients (blue = varus group, red = valgus angle)

The mean WOMAC and KSS of the neutral group were 34.71, 36.61, 33.07, and 36.89. The average WOMAC and KSS in the neutral group were 73.08, 86.19, 72.72, and 87.60 (Table 3). The changes of the varus and valgus angles of all the patients after surgery are shown in Fig. 6.

## Discussion

The main finding of this study is to determine whether the change of soft tissue relaxation around the knee with time after TKA will affect the lower limb alignment as well as whether the lower limb alignment of different degrees after TKA will affect the soft group remodeling.

Rectangular joint space is the goal of achieving satisfactory soft tissue balance because this type of space is conducive to the functional recovery of the knee joint, appropriate contact pressure, and kinematics of the tibiofemoral joint. Asymmetry of the joint space and poor soft tissue balance are recognized causes of instability and poor prognosis after TKA [12].

We strictly controlled the influencing factors during the process of taking the full-length X-ray image of the lower limb weight bearing position in order to ensure that the lower limb is in a neutral position. We used the HKA angle measured one month after the operation to represent the earliest lower limb force line of the patient because the patient could not reach the standard full-length X-ray results of the lower limb in the weight bearing position within one month after the operation due to pain and psychological fear. According to the consensus formed by previous research results, it is considered that the HKA angle is neutral if it does not exceed ± 3° [13]; therefore, we set the HKA angle ≤  ± 3° as the neutral group, and >  ± 3° as the deviation group. According to our results, the lower limb mechanical alignment of the two groups of subjects after surgery did not change significantly over time. The alignment of residual lower limb varus after surgery did not change after 1 year [14]. We believe this is due to two reasons: First, although there is a statistical difference in the change of bilateral soft tissue relaxation, the value is still small enough to cause significant changes in lower limb alignment, and second, the HKA angle we measured was taken under load. The force provided by soft tissue tension was not enough to completely resist its weight. The decisive factor of lower limb alignment was still dominated by bone structure.

Different from the 150N stress used in other previous studies, we applied a 100N stress during the stress test, because we found that most patients could not tolerate the 150N force during our test, and the patients would adjust their positions autonomously when they were in pain, which affects the accuracy of the measurement results. The research of Tetsuro Ushio et. al. believes that using 100N instead of 150N can more accurately measure data and effectively reduce load related pain [11]. It is worth noting that even if we reduce the stress to 100N, a small number of patients will still feel slight pain.

This study found that the change of soft tissue relaxation was most apparent at 3 months after TKA, which may be due to the gradual relief of knee joint pain and soft tissue healing leading to a shift of the patient's body weight to the surgical side [15]. This indicates that soft tissue remodeling was completed 3 months after TKA. Hitoshi Sekiya's research results also proved that 3 months after TKA is the time frame for soft tissue changes to occur [2]. To date, all previous relevant studies did not explore the difference of soft tissue changes between different lower limb alignment groups. In this study, it was found that the relaxation of the medial soft tissue in the neutral and the deviation groups increase with time during the first 3 months after surgery, with the change in the neutral group being greater than that in the deviation group (*p* < 0.05). The relaxation of the lateral soft tissue within the two groups decreased within 3 months after surgery, and there was no significant difference between the two groups. The research demonstrated that the varus alignment hinders early medial soft tissue remodeling, but not lateral soft tissue remodeling. This may be because compared to the neutral group, the tension of the medial collateral ligament in the deviation group is lower [16], which affects soft tissue remodeling. We found that even if the medial soft tissue remodeling of the deviation group was limited, there were still significant changes at 3 months after surgery compared to1 month after surgery. This demonstrates that the lower limb alignment has minimal influence on the soft tissue remodeling. The conclusion is that there is no significant difference between the clinical results of the two groups.

The Hatayama K et. al. study shows that the residual varus limb alignment does not increase lateral relaxation at 5 years of follow-up [14], which is consistent with the short-term results of our study. Sekiya H et. al. believes that the lateral relaxation of patients after surgery will decrease with time, and the medial relaxation will not change significantly [2]. However, Tsukeoka T et. Al’s. measurement results within the patient's anesthesia state shows that the medial relaxation will increase after surgery, and the lateral relaxation will not change significantly [7]. We found that even if the medial soft tissue remodeling was limited in the deviation group, there was no significant difference in the postoperative results. In recent years, robot technology has been advocated because of accurate osteotomy and soft tissue balance. However, if the accuracy of the relaxation parameters does not affect the results, then traditional surgical methods with low cost may have the same value [8].

In a bilateral comparative study, the soft tissue of the knee joint after TKA was more comfortable than the loose and tense sides [17]. The results of this study prove that good postoperative lower limb alignment is more conducive to soft tissue balance, although there is no significant difference in satisfaction. Accurate alignment correction can also reduce the pressure on the medial tibiofemoral compartment, thereby slowing down the wear rate of the medial prosthesis [18]. According to the questionnaire survey, 91% of patients expect an accurate alignment of the lower limbs after surgery. In order to achieve better soft tissue balance and meet the expectations of patients after surgery, we suggest that TKA surgery should achieve accurate alignment (varus ≤ 3°). In addition, previous studies have confirmed that most healthy people are varus within 3° [13].

The ROM of patients after surgery was significantly improved compared with that before surgery, with no significant difference between the two groups, which proved that there was no significant correlation between knee alignment and activity function after surgery and is consistent with previous relevant research reports [19].

Although previous studies have shown that poor postoperative alignment is related to low postoperative satisfaction [19], our study did not find significant differences between the two groups in the WOMAC and KSS scores, which may be due to the maximum varus angle in the deviation group being only 5.6°, and is not enough to support patients to correctly judge their lower limb alignment [20]. The lower limb alignment in the deviation group also has improved significantly compared to before surgery, thus, meeting the needs of patients.

Abdel et. al. followed up 398 patients with TKA for 20 years, and there was no significant difference in TKA revision rate caused by infection, aseptic loosening, mechanical failure, abrasion, patellofemoral trajectory problems between the alignment less than ± 3° group, and alignment greater than the 3° group [21]. However, some former scholars believed that lower limb alignment control within 3° can reduce the wear and looseness of the prosthesis [10, 22]. Although there was no significant difference in functional scores between the two groups in the short term after surgery, medial soft tissue remodeling in the neutral group was greater than that in the deviation group. Furthermore, considering the wear and looseness of the prosthesis, the long-term results need to be further studied. Previous studies have shown that the neutral alignment formed by ipsilateral femoral valgus compensating for tibial varus is the primary morphological mode of healthy middle-aged people [23]. Therefore, the author suggests that the lower limb alignment of patients should be kept within 3° of varus during a TKA operation, so that patients can return to a healthy state.

In this study, all patients underwent unilateral TKA, and it is unknown whether there is any difference in the results of bilateral TKA. Previous studies have found that patients undergoing bilateral TKA at one-stage surgery showed functional results comparable with the results of a unilateral TKA [24].

This paper only studied PS TKA, and whether the results of CR TKA are the same needs further evaluation [25]. A previous study found that the average internal and external relaxation of patients with PS TKA was greater than that of patients with CR TKA, however, the difference was not statistically significant [26]. Another study found that there was no statistical differences between CR and PS knee joint relaxation. Additionally, good results could be obtained by maintaining the knee joint relaxation of approximately 4° [27]. Therefore, we beleive it may be that the posterior cruciate ligament plays a limiting role in the internal and external relaxation [28–30], the cam box of PS TKA cannot increase the valgus stability [31], and the force is not enough to cause a significant difference. Kayani's et. al. research proved that resection of the posterior cruciate ligament will not significantly increase the relaxation of the internal and external soft tissues in the extension position; however, can increase the relaxation of the internal and external soft tissues in the flexion position [29]. In addition, attention should be paid to the impact of BMI on soft tissue relaxation. In patients with lower limb varus, high BMI will make the lateral soft tissue of the knee bear a greater stress [22]. In our study, there was no statistical difference in BMI between the two groups. Different scoring systems may have different results, and recent studies have found that using the Forgotten Joint Score to evaluate TKA postoperative outcomes has more clinical value [32–34].

Previous studies have shown that slight joint space imbalance in TKA surgery is acceptable [1]. Kamenaga et. al. believe that joint relaxation after TKA is only one aspect that affects the prognosis, as well as many other factors that will affect the results [12]. The knee varus does not represent tension of the medial ligament [11] or whether soft tissue release should be carefully evaluated before and during operation.

This paper has the following limitations: 1. This study did not include valgus knee patients, and whether those results are the same as this study’s results needs to be further studied; 2. The follow-up time is short, and the long-term results need to be proven by future research; 3. This paper did not measure the change of soft tissue relaxation during buckling, which needs further study. 4. Only PS-type TKA patients were included in this study. Furthermore, whether CR-type TKA results are consistent needs further investigation.

This study shows that no matter if the lower limb varus alignment correction is accurate during TKA, the tension of internal and external soft tissues can be remodeled during the early postoperative period. The tension change of internal soft tissues in patients with accurate alignment is greater than in patients with inaccurate alignment. Additionally, there is no correlation between the remodeling of external soft tissues and alignment. Postoperative changes in soft tissue balance will not significantly affect lower limb alignment. Therefore, preservation of part of the medial interventricular tension during operation can achieve good soft tissue balance and postoperative results.

## Data Availability

The datasets used or analyzed during the current study are available from the corresponding author on reasonable request.
